# Characteristics and Outcomes of Children, Adolescents and Young Adults with Relapsed/Refractory Non-Hodgkin Lymphoma Undergoing Autologous Stem Cell Transplant

**DOI:** 10.21203/rs.3.rs-2531406/v1

**Published:** 2023-02-07

**Authors:** Yago Nieto, Oren Pasvolsky, Roland Bassett, Sassine Ghanem, Branko Cuglievan, Priti Tewari, CHITRA HOSING, Samer Srour, Jeremy Ramdial, Kris Mahadeo, Sajad Khazal, Demetrios Petropoulos, Uday Popat, Muzaffar Qazilbash, Partow Kebriaei, Richard Champlin, Elizabeth Shpall

**Affiliations:** MD Anderson Cancer Center, University of Texas; University of Texas MD Anderson Cancer Center; The University of Texas MD Anderson Cancer Center; Warren Alpert School of Medicine at Brown University; The University of Texas MD Anderson Cancer Center; The University of Texas MD Anderson Cancer Center; M.D. ANDERSON CANCER CENTER; The University of Texas MD Anderson Cancer Center; M D Anderson Cancer Center; The University of Texas MD Anderson Cancer Center; The University of Texas MD Anderson Cancer Center; The University of Texas MD Anderson Cancer Center; The University of Texas MD Anderson Cancer Center; The University of Texas MD Anderson Cancer Center; MD Anderson Cancer Center; University of Texas MD Anderson Cancer Center; M.D. Anderson Cancer Center

**Keywords:** non hodgkin lymphoma, children, adolescents, young adults, autologous transplant

## Abstract

There is paucity of data regarding outcomes of children, adolescents and young adults (CAYA) patients with non-Hodgkin lymphoma (NHL) undergoing autologous hematopoietic stem cell transplantation (ASCT). We analyzed 222 patients aged 0–39 years undergoing first ASCT for NHL between 2000 and 2020. The most common histological subtypes were DLBCL (44%), T-NHL (19%) and PMBCL (19%). Younger patients (age ≤ 25) had lower incidence of DLBCL and higher incidence of PMBCL and T-NHL compared to older patients (> 25 years) (P = 0.02). None of the younger patients had DH)/DE DLBCL, as compared to 14 patients in the older group (18%, P = 0.07).

Younger patients had numerically better 15-year post-transplant PFS (67% vs. 54%) and OS (71% vs. 62%) compared to older patients, without statistically significant differences (P = 0.19 and P = 0.24, respectively).

In MVA, not achieving a CR prior to ASCT was independently predictive of worse PFS (P < 0.0001). DH/DE status was an independent adverse predictor of PFS in MVA (HR 5.8, p = 0.03). 10 patients(4.5%) (all aged > 25 years) developed SPM

Patients aged ≤ 25 years presented a distinct NHL histology as compared to older CAYA patients. Disease status at ASCT was predictive of both PFS and OS. DH/DE status was an adverse predictor of PFS.

## Introduction

There is growing recognition of the unique characteristics and challenges facing children, adolescents and young adults (CAYA) with cancer (1). NHL accounts for approximately 8% of reported malignancies in the AYA population. AYA with NHL have distinct clinical presentations, biological characteristics and outcomes compared to children or older adults (2). The most common NHL subtypes in this age group include diffuse large B-cell lymphoma (DLBCL), anaplastic large cell lymphoma, Burkitt lymphoma, lymphoblastic lymphoma and primary mediastinal large B-cell lymphoma (PMBCL). Compared to older adults, DLBCL in children and AYA is more likely to have a germinal center B-like (GCB) immunophenotype, *MYC* translocation or overexpression, high proliferative index and absence of BCL2 translocation (3–5).

CAYA patients undergoing hematopoietic stem cell transplantation (HSCT) face unique difficulties, including social and psychological challenges (6, 7), yet there is paucity of data focusing on outcomes of ASCT in CAYA patients with NHL.

In the present study we aimed to examine the characteristics and outcomes of contemporaneous CAYA patients who received their first ASCT due to NHL at our center. We evaluated potential impact of patient and disease-related variables on outcomes and compared the younger and older age groups within our cohort.

## Methods

### Study design and participants

We conducted a retrospective chart review of MD Anderson Cancer Center patients aged 0–39 years who received their first ASCT for NHL between January 2000 and January 2020. We excluded patients who received a previous allogeneic or autologous transplant. We divided this cohort into two age groups: younger (≤ 25 years) and older (> 25 years) patients. This division was based on developmental stage: ≤25 years to include pediatric, adolescents and emerging adults, and > 25 years to include young adulthood patients (8). This retrospective chart review was approved by the Institutional Review Board.

We used standard criteria for staging, response and outcomes according to guidelines from the Lymphoma Imaging Working Group (9) and Harmonization Project in Lymphoma (10). As per these criteria, complete remission (CR) was defined as the disappearance of any evidence of disease on positron emission tomography/computed tomography (PET/CT); partial remission (PR) was defined as regression of measurable disease and no new sites on PET/CT; stable disease (SD) was defined as failure to attain either CR/PR or progressive disease (PD); PD was defined as any new lesion or increase by ≥ 50% of previously involved sites from nadir on PET/CT.

Double/triple hit lymphoma and double expressor lymphoma were defined according to the WHO classification of lymphoid neoplasms (11).

### Statistical methods

Wilcoxon rank-sum tests were used to compare the distribution of continuous variables between age groups. Fisher’s exact tests were used to compare the distribution of categorical variables between groups. The method of Kaplan and Meier was used to estimate the distribution of overall survival (OS) and progression-free survival (PFS) from the date of transplant. Patients who remained alive (OS) or alive and progression-free (PFS) were censored at the last follow-up date. Distributions were compared using the log-rank test. Cox proportional hazards regression models were fit to each survival endpoint. In some cases, Firth’s penalized likelihood method was used to fit models when the number of patients in one or more categories was small. Univariate and multivariate Cox regression models were also fit to each survival endpoint using factors specified by the investigator. Because there was substantial missing data in the second-line international prognostic index (sIPI) variable, two models were fit for each: one considering sIPI and one without it.

All statistical analyses were performed using R version 4.1.1. All statistical tests used a significance level of 5%. No adjustments for multiple testing were made.

## Results

### Patients and disease characteristics

We included 222 consecutive patients in the analysis, 130 (59%) were male and 92 (41%) female. The median age was 32 (range 6–39) years. The most common histological subtypes in the entire cohort were diffuse large B cell lymphoma (DLBCL) (N = 98, 44%), T-NHL (N = 43, 19%) and primary mediastinal B-Cell lymphoma (PMBCL) (N = 41, 19%) (Table 1). Within the subset of DLBCL patients, 14 patients (14%) had either double/triple hit or double expressor lymphoma. Most patients received 2 (37%) or 3 (31%) lines of treatment prior to ASCT, and the majority achieved a pre-transplant response of either CR (59%) or PR (32%).

Younger patients (age ≤ 25) had a trend towards a lower incidence of DLBCL (33% vs. 48%) and higher incidence of PMBCL (24% vs. 17%) and T-NHL (32% vs. 16%) compared to older patients (P = 0.02). None of the younger patients had double hit (DH)/double expressor (DE) DLBCL, compared to 14 patients in the older group (18%, P = 0.07). Number of treatment lines and responses prior to ASCT were similar in both age groups (p = 0.93 and p = 0.71, respectively) (Table 1).

### Outcomes

**Figure 1:**
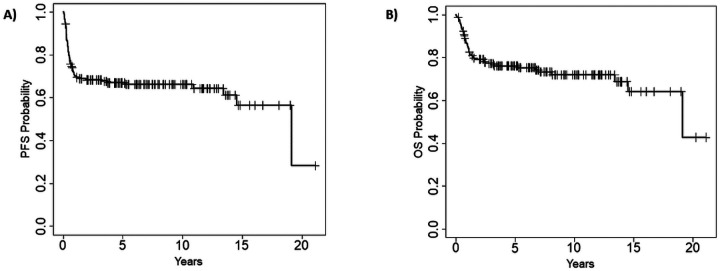
Progression free survival (A) and overall survival (B) for the entire cohort.

**Figure 2:**
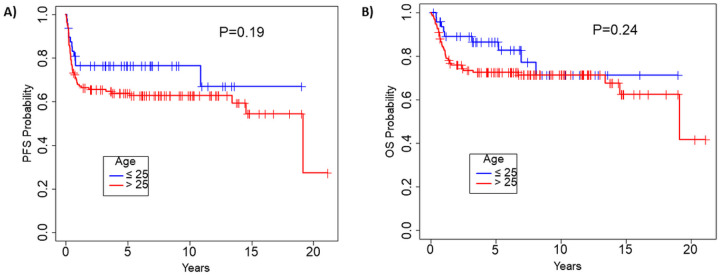
Progression free survival (A) and overall survival (B) according to age at transplant: younger (≤25 years) versus older (>25 years) patients.

After a median follow up of 5.5 (range 0.1–21.1) years, the median PFS for the entire cohort was 19.1 (95% CI 14.5 – NR) years (Fig. 1A) and the median OS was 19.1 (95% CI 19.1 – NR) years (Fig. 1B). When considering the three main aggressive NHL subtypes (DLBCL, PMBCL and T-NHL), younger patients had numerically better 15-year post-transplant PFS (67% vs. 54%, Fig. 2A) and overall survival (OS) (71% vs. 62%, Fig. 2B), as compared to older patients, yet these differences did not reach statistical significance (P = 0.19 and P = 0.24, respectively). The most common cause of death in both age groups was progression of lymphoma, that accounted for 43 deaths (72% of all deaths) (supplementary Table 1).

Number of previous lines of treatment was predictive of PFS in univariate analysis (HR 1.35, p = 0.02), but not multivariate analyses (MVA). In MVA, not achieving a CR prior to ASCT was independently predictive of worse PFS [PR (hazard ratio (HR), 3.9); SD (HR, 18.0), P = 0.03] and of worse OS [PR (HR, 4.2), SD (HR, 6.5) and PD (HR, 4.7), P < 0.0001]. DH/DE status was an independent adverse predictor of PFS in MVA (HR 5.8, p = 0.03), but not of OS (HR = 1.8, p = 0.26). Complete MVA for PFS and OS are provided in Table 2. UVA for PFS and OS are provided in Supplementary Table 2 and Supplementary Table 3, respectively.

Ten patients in the cohort (4.5%), all aged > 25 years, developed second primary malignancies (SPM), at a median of 34.4 (range, 1.0–196.6) months after ASCT: 6 hematological malignancies (myelodysplastic syndrome (MDS) n = 4, acute myeloid leukemia n = 2) and 4 solid tumors (sarcoma, squamous cell carcinoma, thyroid cancer and lung cancer n = 1 each). SPM was the cause of death in five (50%) of them. Three patients with t-MDS received an allogeneic HCT. For all patients with a SPM, median PFS and OS times were both 161.2 (10.2 – NR) months following ASCT.

## Discussion

In the present study we describe patient characteristics and long-term outcomes of a large cohort of CAYA who received ASCT for NHL at our center. The three most common histological subtypes of NHL in the entire cohort were DLBCL, T-NHL and PMBCL. More than 90% achieved ≥ PR prior to transplant. We found that patients aged ≤ 25 years presented a distinct NHL histology as compared to older CAYA patients, and none in this younger age group had DH/DE DLBCL. Disease status at ASCT was predictive of both PFS and OS, whereas DH/DE status was an adverse predictor of PFS.

We observed numerically better survival outcomes in younger patients, yet there was no statistically significant difference between the two age groups. A report by Berkman et al. examined long-term survivors of DLBCL AYA patients (defined as age of 15–39 years) using the Surveillance Epidemiology and End Results (SEER) database (12) found that each additional year of age at diagnosis was associated with a decrease of 6% in overall survival (p < 0.0001). On the other hand, in the Japanese AYA registry study (13), age was not associated with survival outcomes. In the subset of patients who received ASCT, younger patients (< 16 years) had similar OS and EFS compared to older patients (≥ 16 years) [(45.7% vs. 44.8%, p = 0.547) and (42.3% vs. 38.9%, p = 0.775), respectively]. However, younger patients in that study did have a higher rate of transplant related mortality (TRM) (5.1% vs. 0.8%, p = 0.0043). We observed higher rates of PFS and OS in our cohort, compared to the Japanese registry study: The 5-year PFS in our study was 76.5% for the younger age group and 63.8% in the older age group, and the 5-year OS were 86.4% and 72.5%, respectively. There are several methodological differences that can partially explain these differences in outcomes. First, we included a broader range of ages in our study, so that our study cohort comprised both pediatric patients as well as the most commonly used definitions of AYA, up to the age of 39 (6). Furthermore, the two studies also had different proportions of the various NHL histological subtypes and used a different age cutoff for the younger/older age subsets.

Few previous reports have compared outcomes of ASCT to allogeneic HSCT in NHL with a varying range of ages within the CAYA spectrum (13–16). Most of these reports showed similar outcomes between the two types of HSCT, including a small single center study that included 36 pediatric NHL patients (15) and a Center for International Blood and Marrow Transplant Research (CIBMTR) registry study that included 182 children and adolescent NHL patients (aged 0–18 years; 90 received ASCT) that were transplanted between 1990 and 2005 (16). A more recent analysis of the Japanese registry dataset compared outcomes of AYA patients (defined as aged 16–30, n = 645) to pediatric patients (aged 0–15, n = 273) undergoing autologous or allogeneic stem cell transplant for NHL (13). Outcomes were similar between the two age groups among those who received an allogeneic transplant. However, as previously mentioned, TRM in the 55% of the cohort that received ASCT was significantly higher in children compared to AYA. A multi-national study included 639 patients with R/R NHL (mostly Burkitt lymphoma/leukemia and DLBCL) aged 0–18 years. Only 23% of the entire cohort (n = 150) received an ASCT, whereas 39% received allogeneic transplant and 37% did not undergo any transplant. The 8-year OS of patients who underwent ASCT was superior to those who received an allogeneic transplant or did not receive any transplant (55% vs.47% vs. 8%, respectively; p < 0.0001).

Our study focused only on first ASCT, and included both the pediatric and AYA populations. We were able to elucidate variables that impacted survival outcomes in this setting. We showed that achieving pre-transplant CR was associated with better PFS and OS in MVA. This finding complements previous studies that have shown the prognostic impact of responses prior to ASCT in adults with aggressive B-Cell (17) and T-Cell (18) NHL.

In our cohort none of the younger DLBCL patients had DH/DE lymphoma, whereas 14% of patients aged > 25 years had either DH or DE disease. A previous study also observed that patients with DH/DL DLBCL were overall older than those without one of these high-risk features, and the youngest patients with DH and DE were 49 and 30 years of age, respectively (19). Of note, patient age in that study ranged between 16–91 (median 64) years. In another series, only one of 16 pediatric DLBCL patients had DH DLBCL (3). In the current study we confirmed the prognostic importance of DH/DE status in CAYA patients undergoing ASCT. A previous report demonstrated the prognostic impact of DH/DE status in adults (age range 30–76 years) (20). To the best of our knowledge, we are the first to report this finding in the CAYA DLBCL population.

With a median follow up period of 5.5 years, and some patients followed for more than 20 years, 4.5% of our CAYA cohort develop a SPM. This is lower than the 9% of SPM reported in a study that included 372 adult NHL patients who underwent ASCT (21), and slightly higher than the 2.6% reported in a large cohort of 1487 pediatric patients that were transplanted for a variety of indications (26% underwent ASCT due to lymphoma) (22). A joint study from European and German study groups analyzed 189 cases of SPM in children and adolescents (aged 0–18) after treatment for NHL (23). Half the SPM in that study were hematological malignances (23% myeloid and 27% lymphoid neoplasms), and an additional 25% were carcinomas. This distribution was overall similar to the types of SPM observed in our cohort. Of note, the aforementioned study did not report how many patients received ASCT.

The current study has several limitations inherent to its retrospective design, including heterogeneity in patient characteristics and treatments, as well as unidentified confounders that were not accounted for, despite the use of multivariable cox regression analysis. Furthermore, cohort size is rather modest, despite being one of the largest cohorts of CAYA NHL patients receiving ASCT in the published literature to date.

In conclusion, we described patient characteristics and long-term outcomes of a large cohort of CAYA who received ASCT for NHL. Patients aged ≤ 25 years presented a distinct NHL histology as compared to older CAYA patients, and none in this younger age group had DH/DE DLBCL. We observed numerically better PFS and OS in younger patients, albeit without a statistically significant difference. Disease status at ASCT was predictive of both PFS and OS. DH/DE status was an adverse predictor of PFS.

With the advent of novel therapeutic modalities, mainly CAR T therapy, the role of ASCT needs additional clarification. Recently, two phase III trials have shown superiority of two anti-CD19 CAR T constructs for treatment of a subset of patients with R/R DLBCL (24, 25). Both trials included only adult patients, and trials with autologous anti-CD19 CAR T in pediatric NHL patients are ongoing (NCT03610724, NCT02625480). Our data may serve as a benchmark for outcomes of future CAR T trial outcomes.

## Figures and Tables

**Table 1:** Patient and disease characteristics

Parameter	All (N = 222)	Age ≤ 25 years (N = 54)	Age > 25 years (N = 168)	p-value
	n (%)	n (%)	n (%)	
**Gender**				1.0
Male	130 (59)	32 (59)	98 (58)	
Female	92 (41)	22 (41)	70 (42)	
**Histology**				0.05
DLBCL	98 (44)	18 (33)	80 (48)	
T-NHL	43 (19)	17 (32)	26 (16)	
PMBCL	41 (19)	13 (24)	28 (17)	
Low-grade B-Cell lymphoma	10 (5)	0 (0)	10 (6)	
Hepatosplenic gamma-delta T-cell lymphoma	7 (3)	2 (4)	5 (3)	
Burkitt lymphoma	7 (3)	2 (4)	5 (3)	
Primary CNS Lymphoma	4 (2)	0 (0)	4 (2)	
Plasmablastic lymphoma	3 (1)	0 (0)	3 (2)	
Lymphoblastic lymphoma	3 (1)	0 (0)	3 (2)	
Mantle Cell Lymphoma	2 (1)	0 (0)	2 (1)	
Waldenstrom’s macroglobulinemia / lymphoplasmacytic lymphoma	1 (0)	0 (0)	1 (1)	
Small noncleaved cell, non-Burkitt lymphoma	1 (0)	1 (2)	0 (0)	
Hodgkin lymphoma, lymphocyte predominant	1 (0)	1 (2)	0 (0)	
Cutaneous T-cell lymphoma/Sezary	1 (0)	0 (0)	1 (1)	
**Double or Triple Hit / Double Expressor status** ^¥^				0.07
Positive	14 (14)	0 (0)	14 (18)	
Negative	84 (86)	18 (100)	66 (83)	
**No. treatment lines before ASCT**				0.93
1	42 (19)	9 (17)	33 (20)	
2	82 (37)	21 (39)	61 (36)	
3	69 (31)	19 (35)	50 (30)	
4	22 (10)	4 (7)	18 (11)	
5	7 (3)	1 (2)	6 (4)	
**Response prior to ASCT**				0.71
CR	131 (59)	36 (67)	95 (57)	
PR	72 (32)	15 (28)	57 (34)	
SD	9 (4)	1 (2)	8 (5)	
PD	9 (4)	2 (4)	7 (4)	
Untreated	1 (0)	0 (0)	1 (1)	
**sIPI**				0.55
0/1	84 (82)	22 (88)	62 (81)	
2/3	18 (18)	3 (12)	15 (20)	
**Conditioning regimen**				0.55
BEAM +/− rituximab	138 (62)	37 (69)	101 (60)	
GemBuMel - based	71 (32)	14 (26)	57 (34)	
**KPS/LPS prior to transplant**				0.62
100	63 (33)	15 (32)	48 (33)	
90	90 (47)	22 (47)	68 (47)	
80	32 (17)	8 (17)	24 (16)	
70	7 (4)	1 (2)	6 (4)	
60	1 (1)	1 (2)	0 (0)	

DLBCL = diffuse large B-cell lymphoma; T-NHL = T-cell non-hodgkin lymphoma; PMBCL = primary mediastinal B-cell lymphoma; CNS = central nervous system; ASCT = autologous hematopoietic stem cell transplantation; CR = complete remission; PR = partial remission; SD = stable disease; PD = progressive disease; sIPI = secondary international prognostic index; BEAM = BCNU, etoposide, cytarabine and melphalan; GemBuMel = gemcitabine, busulfan and melphalan; KPS/LPS = karnofsky/lansky performance scale.

¥ Considered positive if either of the following positive: double hit / triple hit / double expressor. Calculated for patients with DLBCL histology only.

**Table 2:** Multivariable Assessments for progression free survival (PFS) and overall survival (OS).

Double or Triple Hit / Double Expressor status^¥^		
PFS		
Parameter	Hazard Ratio (95% CI)	p-value
Negative	*ref*	
Positive	5.8 (1.2–23.3)	**0.03**
**Response prior to ASCT**		**0.03**
CR	*Ref*	
PR	3.9 (1.2–13.3)	
SD	18.0 (1.3–178.3)	
**Number of treatment lines prior to ASCT**	0.8 (0.4–1.6)	0.62
**sIPI**		
0/1	*Ref*	
2/3	0.7 (0.2–2.6)	0.63
**Conditioning Regimen**		
BEAM +/− rituximab	*Ref*	
GemBuMel-based	0.5 (0.1–1.9)	0.31
**OS**		
**Response prior to ASCT**		**<0.0001**
CR	*ref*	
PR	4.2 (2.2–8.7)	
SD	6.5 (1.9–18.5)	
PD	4.7 (0.9–17.2)	
**Lines of treatment prior to ASCT**	1.1 (0.8–1.5)	0.45
**Conditioning Regimen**		
BEAM +/− rituximab	*ref*	
GemBuMel-based	1.0 (0.5–1.8)	0.93

**Abbreviations:** DH/DE= double hit/double expressor; ASCT = autologous hematopoietic stem cell transplantation; CR = complete remission; PR = partial remission; SD = stable disease; PD = progressive disease; sIPI = secondary internation prognostic index; BEAM = BCNU, etoposide, cytarabine and melphalan; GemBuMel = gemcitabine, busulfan and melphalan.

¥ Considered positive if either of the following positive: double hit / triple hit / double expressor.Calculated for patients with DLBCL histology only.
